# Cutaneous sensory symptoms and emotional regulation in non-clinical healthy students: a near-infrared spectroscopy study

**DOI:** 10.3389/fpsyg.2025.1619280

**Published:** 2025-09-22

**Authors:** Sachiyo Ozawa, Hiromasa Yoshimoto, Hajime Tanabe, Shinsuke Koike

**Affiliations:** ^1^Faculty of Sociology, Department of Social Psychology, Toyo University, Tokyo, Japan; ^2^Institute of Industrial Science, The University of Tokyo, Tokyo, Japan; ^3^Faculty of Humanities and Social Sciences, Shizuoka University, Shizuoka, Japan; ^4^Center for Evolutionary Cognitive Sciences, Graduate School of Arts and Sciences, The University of Tokyo, Tokyo, Japan

**Keywords:** mental stress, emotional distraction, mind-wandering, task-unrelated thoughts, Sustained Attention to Response Tasks (SART), near-infrared spectroscopy (NIRS), medial prefrontal cortex(mPFC)

## Abstract

**Background:**

In an era of increasing societal stress, greater attention must be given to the mental health and well-being of non-clinical populations. Many individuals in these groups report cutaneous sensory symptoms—such as pain, crawling sensations, and burning—linked to underlying mental stress. To investigate the relationship between these stress indicators and well-being, this study examines whether university students with higher versus lower levels of these symptoms differ in their ability to regulate emotions through distraction.

**Methods:**

Data were collected from 57 university students (mean age: 19.26 ± 1.03 years, range: 18–23 years, 22 males), including near-infrared spectroscopy (NIRS) data from 53 participants. Students were categorized into higher (*n* = 21) and lower (*n* = 36) cutaneous symptom groups based on the mean Cutaneous9 score. In the experiment, the participants first rated their Cutaneous9 symptoms, then completed an emotion-induction task by recalling stressful interpersonal events. Next, they performed the Sustained Attention to Response Tasks (SART) as a distraction. The SART is a type of go/no-go task that includes spontaneous thought probes at pseudo-random intervals to assess attentional state or degree of off-task thoughts. Prefrontal activity during the SART was assessed using NIRS. Emotional states were assessed before induction, after induction, and after distraction using the Positive and Negative Affect Schedule.

**Results:**

Participants with higher cutaneous symptoms struggled more with maintaining attention during SART and showed less reduction in unpleasant emotions. Only those with lower symptoms experienced a decrease in unpleasant emotions. Furthermore, participants with higher cutaneous symptoms exhibited medial prefrontal cortex activation during distraction, with no significant activation differences in the lateral prefrontal cortex.

**Conclusion:**

These findings suggest that university students with higher cutaneous symptoms face greater challenges in regulating emotions compared with those with lower symptoms. Their reduced ability to benefit from distraction may stem from difficulties in maintaining attentional focus or increased absorption in off-task thoughts, rather than impaired emotional inhibition. Addressing these challenges through targeted interventions may enhance the mental well-being and overall quality of life in non-clinical populations.

## 1 Introduction

Clinical psychology has traditionally focused on the mental health of patients with psychiatric disorders. However, in today’s increasingly stressful society, there is a growing need to address the psychological health and well-being of non-clinical healthy populations (Huppert, 2009; Trudel-Fitzgerald et al., 2019; Querstret et al., 2020). Although the signs of mental dysregulation in this group may be subtle or implicit, they may still experience distress owing to various mental and physiological factors. To prevent the development of more serious issues and promote overall well-being, it is essential not to overlook signs of mental dysregulation in non-clinical populations and to offer guidance for enhancing their mental health and well-being.

People face various psychological stressors in their daily lives and attempt to cope with them consciously and unconsciously. However, excessive stress and dysregulation of mental processing can lead to dysfunction of the autonomic nervous system. This can result in various psychosomatic symptoms such as headaches, abdominal pain, palpitations, and fatigue, which are often difficult to diagnose medically. According to Gupta and Gupta (2004), cutaneous sensory symptoms not diagnosed as cutaneous disorders, such as pain, burning, crawling sensations, tingling, numbness, and itching, are indicators of psychological stress. These are considered as somatization symptoms (Gupta, 2006) and are related to somatoform dissociation (conversion symptoms; Gupta and Gupta, 2004). Gupta and Gupta (2004) found that such cutaneous sensory symptoms are experienced in non-clinical community samples with no history of dermatological or other medical disorders, and demonstrated that stressful major life events are associated with a higher frequency of symptoms.

People attempt to cope with daily psychological stress and unpleasant emotions by using various emotion regulation methods. One such method is emotional distraction, which diverts attention from unpleasant situations or undesirable emotions (Nolenhoeksema and Morrow, 1991; Stone and Neale, 1984; Thiruchselvam et al., 2012). Common daily emotional distractions include walking, playing sports, watching movies, and reading. Emotional distraction, when adaptively used (Wolgast and Lundh, 2017), helps prevent persistent depressive mood, thereby assisting individuals in coping with daily psychological stress (Nolenhoeksema and Morrow, 1993). However, individuals with underlying mental stress may have more difficulty managing unpleasant emotions; conversely, difficulty in managing these emotions may be related to the presence of such stress. Based on these findings, we hypothesized that the effects of emotional distraction would be reduced in individuals with cutaneous sensory symptoms.

Emotional distraction underlies the reorientation of attentional deployment, and neuroimaging studies have demonstrated its effects using experimental paradigms that focus on attentional reorientation (Kanske et al., 2011; McRae et al., 2010; Ozawa and Hiraki, 2017; Schönfelder et al., 2014; Thiruchselvam et al., 2012; Van Dillen et al., 2009). These paradigms typically involve both emotional stimulation and attention-demanding distraction tasks, followed by an assessment of emotional state. Attention-demanding tasks used for attentional reorientation are often cognitive in nature, such as mental calculations (Kanske et al., 2011; Schönfelder et al., 2014; Van Dillen et al., 2009), verbal or special working memory tasks (McRae et al., 2010; Thiruchselvam et al., 2012), and thinking about something else (Kalisch et al., 2006). In a previous study, we demonstrated that even simple visuomotor tapping tasks that require less attention can reduce the intensity of unpleasant emotions (Ozawa et al., 2022, 2024). In the present study, we assumed that individuals with cutaneous sensory symptoms may have difficulty attending to such simple tasks, which would be related to the difficulty in diverting attention away from unpleasant emotions.

When individuals are not focused on ongoing tasks, their attention may shift from external stimuli to internal processes, causing their minds to wander and generate various thoughts (Gusnard and Raichle, 2001; Mason et al., 2007; Smallwood and Schooler, 2006), which is similar to what occurs in a resting (default mode) state (Gusnard and Raichle, 2001; Mason et al., 2007; Smallwood and Schooler, 2006). These thoughts are typically self-referential and unrelated to external task demands, and are known as task-unrelated thoughts (TUTs) (Giambra, 1995; Shaw and Giambra, 1992). In contrast, sustained and effective concentrations of attention-demanding tasks can reduce mind-wandering or the generation of TUTs and decrease brain activity relative to the resting baseline, a phenomenon known as task-induced deactivation (Binder et al., 1999; Shulman et al., 1997). Based on these findings, we hypothesized that performing attention-demanding tasks as distractions would reduce the frequency of TUTs and induce task-induced deactivation. Notably, TUTs and mind-wandering can serve as distractions by shifting attention away from ongoing tasks, generating positive emotions (Franklin et al., 2013). However, we posited that successfully diverting attention from unpleasant emotions is linked to maintaining sustained task-focused attention, the state not generating TUTs.

The lateral prefrontal cortex (LPFC) areas, including the dorsolateral prefrontal cortex (DLPFC), are typically involved in the cognitive control of emotions (Dolcos et al., 2011; Northoff et al., 2000). Numerous neuroimaging studies have demonstrated that performing attention-demanding cognitive distraction tasks activates the regions, including the DLPFC (Kanske et al., 2011; McRae et al., 2010; Van Dillen et al., 2009). In contrast, the medial prefrontal cortex (MPFC) appears to be associated with various cognitive and emotional functions. The region involves both cognitive control of emotions (Diekhof et al., 2011; Nelson et al., 2015) and emotion generation (Ochsner et al., 2009; Phan et al., 2002). Furthermore, it is well known as a part of the default-mode network and for its association with various cognitive functions, including mind-wandering (Mason et al., 2007) and self-referential thoughts (Gusnard et al., 2001). Christoff et al. (2009) found that MPFC activation was associated with increased rates for self-reports of mind-wandering and performance errors of attention-demanding tasks through a functional magnetic resonance imaging study. Cathodal (inhibitory) transcranial direct current stimulation to the MPFC has been found to reduce mind-wandering (Bertossi et al., 2017; Giacometti Giordani et al., 2023). Patients with lesions to the ventromedial prefrontal cortex have been found to exhibit lower mind-wandering rates during cognitive tasks and report less frequent daydreaming than healthy individuals and controls with lesions not involving the vmPFC (Bertossi and Ciaramelli, 2016). Thus, the MPFC involves various cognitive and emotional processes and is noted to be functionally dissociated from the LPFC (Northoff et al., 2000, 2004). Based on these findings, we hypothesized that effective emotional distraction from unpleasant emotion is primarily associated with the activation of LPFC regions rather than MPFC regions, whereas ineffective emotional distraction is primarily associated with the activation of MPFC regions rather than LPFC regions.

In the present study, we investigated whether the effects of distraction from unpleasant emotion differ between non-clinical participants with higher and lower levels of cutaneous sensory symptoms. We focused on healthy undergraduate students without psychiatric, neurological, or dermatological diseases, assuming that many may experience underlying mental stress and face challenges in emotional management. We hypothesized that participants with more cutaneous symptoms would experience fewer distraction effects. They were indicated by reduced attentional focus on distraction tasks, increased MPFC activation, reduced LPFC activation during distraction, and higher intensity of unpleasant emotions after distraction compared with participants with lower cutaneous symptom levels. To induce unpleasant emotions, we used a paradigm from our previous studies (Ozawa, 2021; Ozawa et al., 2020, 2022), that involved recalling stressful daily interpersonal events. This paradigm was designed to evoke complex social emotions similar to those encountered in real life. We measured the cerebral blood flow (CBF) changes in the PFC using near-infrared spectroscopy (NIRS). It offers quick setup, high tolerance to body movements, and the ability to minimize participant stress during measurements.

First, we investigated whether emotional changes differed between participants with higher and lower cutaneous symptoms following the emotional induction and distraction tasks. Second, we examined whether the overall behavioral responses and PFC activation during distraction tasks differed between the two groups. Finally, we assessed whether the groups differed in changes in behavioral responses and PFC activation during the distraction tasks.

## 2 Materials and methods

### 2.1 Participants

This study conformed to the principles of the Declaration of Helsinki and was approved by the University of Tokyo (approval number: 693-4). Participants were recruited through advertisements (e.g., on university bulletin boards) and were preliminarily informed that they would be asked to recall unpleasant events. The advertisements described people who were eligible to participate in the study as those who were native Japanese speakers, right-handed, and healthy, with no history of psychiatric or neurological diseases. Right-handedness was assessed using the Edinburgh Handedness Inventory (Oldfield, 1971). Health conditions were also assessed using a simple questionnaire, which is described later. All participants except for one were students at the University of Tokyo. Each participant received a comprehensive explanation of the experimental procedure and provided written informed consent. Data were collected from 61 participants who agreed to participate. Among these, four participants reported having medically diagnosed cutaneous symptoms on the Cutaneous9, which assesses stress-related cutaneous sensory symptoms (Gupta and Gupta, 2004), and were excluded from the analysis. The remaining 57 participants’ data (mean age: 19.26 ± 1.03 years; range: 18–23 years; 22 males) were divided into higher and lower Cutaneous9-score groups as described later (higher group: 19.48 ± 1.08 years; range: 18–23 years; 7 males, 14 females; lower group: 19.14 ± 0.99 years; range: 18–22 years; 15 males, 21 females)^1^. For NIRS data analysis, data from one additional participant were removed owing to measurement failure, and three others were excluded owing to artifacts, resulting in NIRS data from 53 participants for analysis (19 in the higher group and 34 in the lower group). The participants recruited in this study were not included in any of our previous studies.

### 2.2 Experimental procedures

The experiments were conducted in a shielded room. Each participant sat on a chair approximately 65 cm away from a 19-inch monitor (39 × 29 cm). Before beginning the procedure, the participants received a thorough explanation of the experiment and provided written informed consent. They were informed that they would be asked to recall stressful interpersonal events in their daily lives, but would not be required to share the content of these memories afterward. The participants were asked to avoid unnecessary head and body movements and deep breathing during the NIRS measurements. They were also informed that they would be monitored by an experimenter via video and that they could withdraw from the study at any time by raising their hands without fear of reprisal or negative consequences.

Participants initially completed the Edinburgh Handedness Inventory and a simple questionnaire on their physical and mental conditions. A simple questionnaire was administered to confirm the eligibility of the participants and included the following questions: “Are you currently in a state of mental health such as depression and anxiety?” (*Yes/No*); “Have you been diagnosed with a mental disorder?” (*Yes/No*), “What is your current mental and physical condition?” (1 = *not good*, 2 = *slightly not good,* 3 = *neutral*, 4 = *slightly good, 5* = *very good*), and “How many hours did you sleep last night?” Prior to task, we confirmed that none of the participants responded “*Yes*” for first and second questions, “*not good*” for the third question, or took less than 4-h sleep. Finally, participants responded to Cutaneous9.

Subsequently, the experimenter explained the task procedures, attached NIRS probes (Spectratech OEG-16H; Spectratech Inc., Yokohama, Japan) to the participant’s head, dimmed the room lighting, and conducted calibration. Participants were then asked to engage in a few practice trials to familiarize themselves with the experimental task procedure and key-presses using only their right index finger.

**Figure 1:**
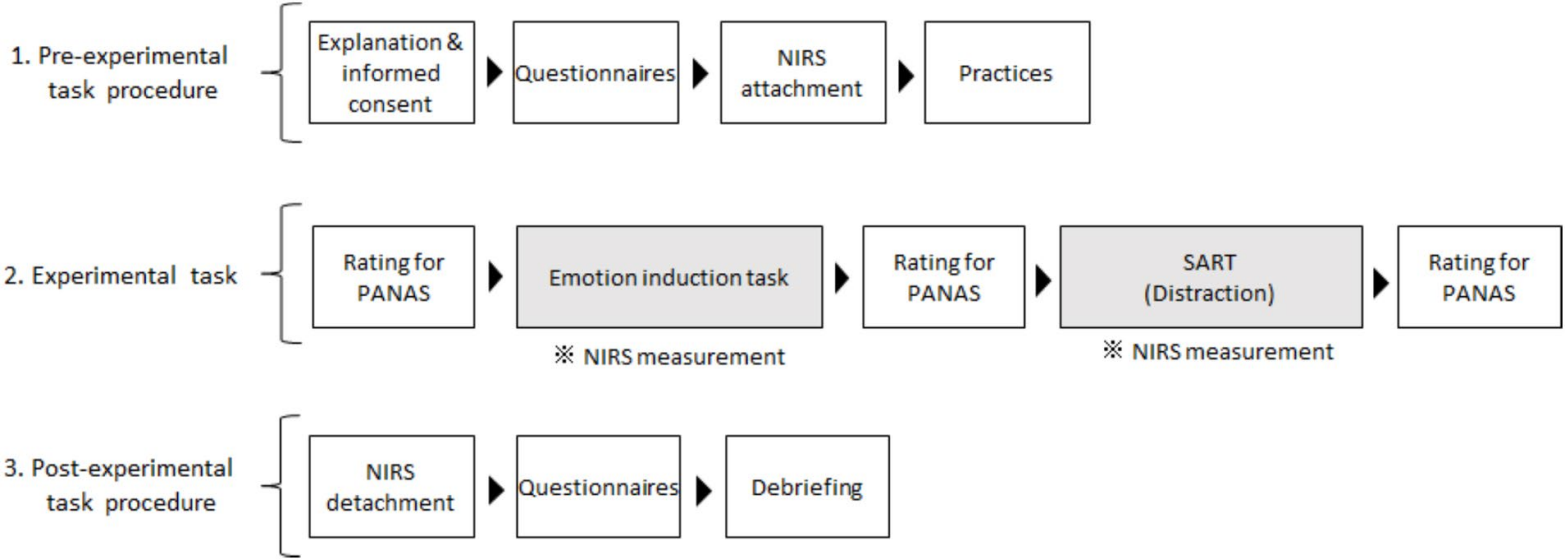
Flow chart of the experimental procedures. Emotional changes were examined across emotion induction and distraction tasks. Prefrontal activities during emotional induction and distraction tasks were measured independently, and those for distraction were assessed in the present study. We measurement prefrontal activities during the emotional induction task and performed questionnaires in the post-experimental task for other research aims. SART, Sustained Attention to Response Tasks; NIRS, near-infrared spectroscopy; PANAS, Positive and Negative Affect Schedule.

Next, participants completed the Positive and Negative Affect Schedule (PANAS; Sato and Yasuda, 2001; Watson et al., 1988) to assess their current emotional state in a well-lit room. Once the room light was dimmed, the participants performed an emotion induction task that involved recalling stressful interpersonal events in their daily lives using our paradigm (Ozawa, 2021; Ozawa et al., 2020, 2022). The PANAS was administered immediately after this task. Next, the participants performed Sustained Attention to Response Tasks (SART; Robertson et al., 1997) as a distraction. After performing the SART, the PANAS was readministered (Figure 1).

At the end of the experiment, the researcher removed the NIRS probes from the head. Additional questionnaires were provided for other research purposes. The experimenter then asked the participants about the emotions they recalled during the experimental task without inquiring about specific memory details. Additionally, participants were asked about aspects of their daily lives that they found enjoyable, such as extracurricular activities, hobbies, and weekend experiences.^2^

### 2.3 Questionnaires

#### 2.3.1 Cutaneous9

Cutaneous9 is a self-report measure that assesses stress-related cutaneous sensory symptoms (Gupta and Gupta, 2004). The nine cutaneous symptoms included in this scale were selected based on common stress-mediated cutaneous complaints reported in the literature and clinical observations by the authors (Gupta and Gupta, 2004). The Cutaneous9 rates the intensity of these nine cutaneous symptoms across 22 body regions on a 10-point scale (0: *not at all*, 9: *very*) over the past month. These symptoms include “burning,” “crawling sensations,” “tingling,” “pricking or pins and needles,” “pain,” “tender to touch,” “numbness,” “moderate to severe itching,” and “easy bruising.” The assessed body regions cover areas of “scalp,” “face,” “lips,” “tongue,” “inside of mouth,” “front of neck,” “back of neck,” “shoulders,” “upper back or area between shoulder blades,” “chest,” “arms,” “forearms,” “hands in general,” “palm(s) of hand,” “lower back,” “abdomen or stomach,” “groin area,” “hips,” “thighs,” “legs,” “feet in general,” and “sole(s) of feet.” Each symptom score was calculated as the sum of the ratings across the 22 regions (range: 0–198), and the total Cutanesou9 score was the sum of the nine symptom scores (range: 0–1782). Cutaeous9 was validated through positive correlations with major life events (*r* = 0.40; *p* < 0.001) and psychological stress (*r* = 0.35; *p* < 0.001) (Gupta and Gupta, 2004), after controlling for psychopathological factors in non-clinical adults with no history of significant dermatological or other medical issues. Additionally, Cutaneous9 has shown a positive correlation with the Dissociative Experiences Scale (DES; Bernstein and Putnam, 1986) in non-clinical populations (Gupta and Gupta, 2004), and it is thought that these sensory symptoms are a form of somatoform dissociation (conversion symptoms).

The Japanese version of the Cutaneous9 asks participants to rate the intensity of nine cutaneous symptoms experienced over the past month on a 10-point scale and to indicate the affected regions by selecting *yes* (1) or *no* (0) for each symptom (Ozawa et al., 2016). Participants were also asked whether they had previously received a medical diagnosis for any of the reported symptoms; if so, their data were excluded from the analysis. The item score for each symptom was calculated by multiplying the symptom intensity score by the number of regions affected (range: 0–198), and the total Cutanous9 score was the sum of all item scores (range: 0–1782). The Japanese version of the Cutaneous9 has demonstrated positive correlations with measures of psychological dissociation assessed by the DES, somatoform dissociations assessed by the Somatoform Dissociation Questionnaire (Nijenhuis et al., 1996), and self-reported childhood abuse history assessed by the Child Abuse and Trauma Scale (Sanders and Becker-Lausen, 1995) by Ozawa et al. (2016).

#### 2.3.2 PANAS

The PANAS is a widely used self-report measure of emotional state (Watson et al., 1988). It includes two scales, Negative Affect (NA) and Positive Affect (PA), each consisting of eight items. The NA scale reflects subjective distress and dissatisfaction associated with negative mood states, including items of “jittery,” “scared,” “upset,” “afraid,” “nervous,” “distressed,” “ashamed,” and “irritable.” The PA scale measures the extent to which a person feels enthusiastic, active, and alert with items of “active,” “proud,” “strong,” “inspired,” “determined,” “excited,” “alert,” and “enthusiastic.” Each item is rated on a 6-point scale from 1 (*not at all*) to 6 (*very*). The PANAS NA or PA score is calculated by summing these item scores. We assessed participants’ emotional states during the experiment using the Japanese version (Sato and Yasuda, 2001), which demonstrated good internal consistency with Cronbach alphas of 0.98 and 0.99 for the PANAS NA and PA, respectively (Sato and Yasuda, 2001).

### 2.4 Emotion induction task

To induce emotions, participants were instructed to recall memories of stressful interpersonal events in their daily lives using our experimental paradigm (Ozawa, 2021; Ozawa et al., 2020, 2022). In this task, a series of instructions and questions was presented on a monitor using white Japanese characters on a black background. Participants could move through each screen by pressing a key at their own pace, except during the baseline and free recall periods, which were set to 15 and 60 s, respectively. The participants were instructed to proceed to the next screen only after fully understanding the instructions or responding sufficiently to the questions.

At the first stage of this procedure, participants were presented with a one-item unpleasant emotion question (“How unpleasant do you feel now?”) and responded on a 7-point scale (1 = *not at all*, 7 = *very*) by pressing a key. A monitor then displayed the following instructions for a 15-s baseline: “Take a brief break. The instructions will be displayed soon (waiting for a while before pressing the key).” Subsequently, a series of instructions was presented, including instructions like “You will be asked to recall stressful interpersonal events in daily life as clearly and vividly as possible” and “you will not be asked for the recalled contents.” Each instruction appeared simultaneously when participants pressed a key. Next, a 60-s free recall period began, displaying the instruction, “Recall while viewing this screen (wait a while before pressing the key).” The participants were able to recall multiple stressful incidents during this time. After the free recall, they received a few additional instructions and questions to guide them to focus on a single stressful incident for the next stage (preliminary question period), with prompts such as “Were you able to remember a stressful experience?,” “When did the incident occur?,” and “You will be asked about the experience shortly.” The participants then responded to a series of questions regarding the details of their behaviors and emotions and other persons involved in the event (response period). These questions included: “What did the person do to you?,” “What did you do to the person?,” “What kind of emotion did you feel?,” “How did you feel about the person?,” “How did you feel about yourself?,” “Did the person know your feelings?,” and “What did you think the person thought about you?” These questions ended with the final instruction, “Please recall the emotions you experienced at that time again.” These questions were designed to enhance memory recall, considering that writing, which is commonly used for autobiographical recall (Dunn and Schweitzer, 2005; Strack et al., 1985), can elicit artifacts in NIRS data due to body movements. After the response period, participants were asked to rate the unpleasant emotion items again. The average time between pre- and post-ratings for the unpleasant emotion item was 372 s. The details and findings of this emotion-induction procedure have been described in our previous studies (Ozawa, 2021; Ozawa et al., 2020, 2022).

### 2.5 SART

For the distraction task, we employed SART (Robertson et al., 1997), which is a type of go/no-go task where participants are required to respond or not to a stimulus (Cheyne et al., 2006; Manly et al., 1999; Robertson et al., 1997). While distraction tasks are generally more complex cognitive tasks, such as mental calculations and working memory tasks, we considered this simpler task suitable for evaluating attentional states and the extent of TUT occurrences during the task. Although the SART demands less attention and may induce fewer attentional reorientations or distraction effects, we assumed that its low difficulty would lead to greater variability in the participants’ ability to focus. This variability would allow for an examination of the differences in distraction effects between participants with higher and lower Cutaneous9 scores in non-clinical, healthy individuals.

**Figure 2:**
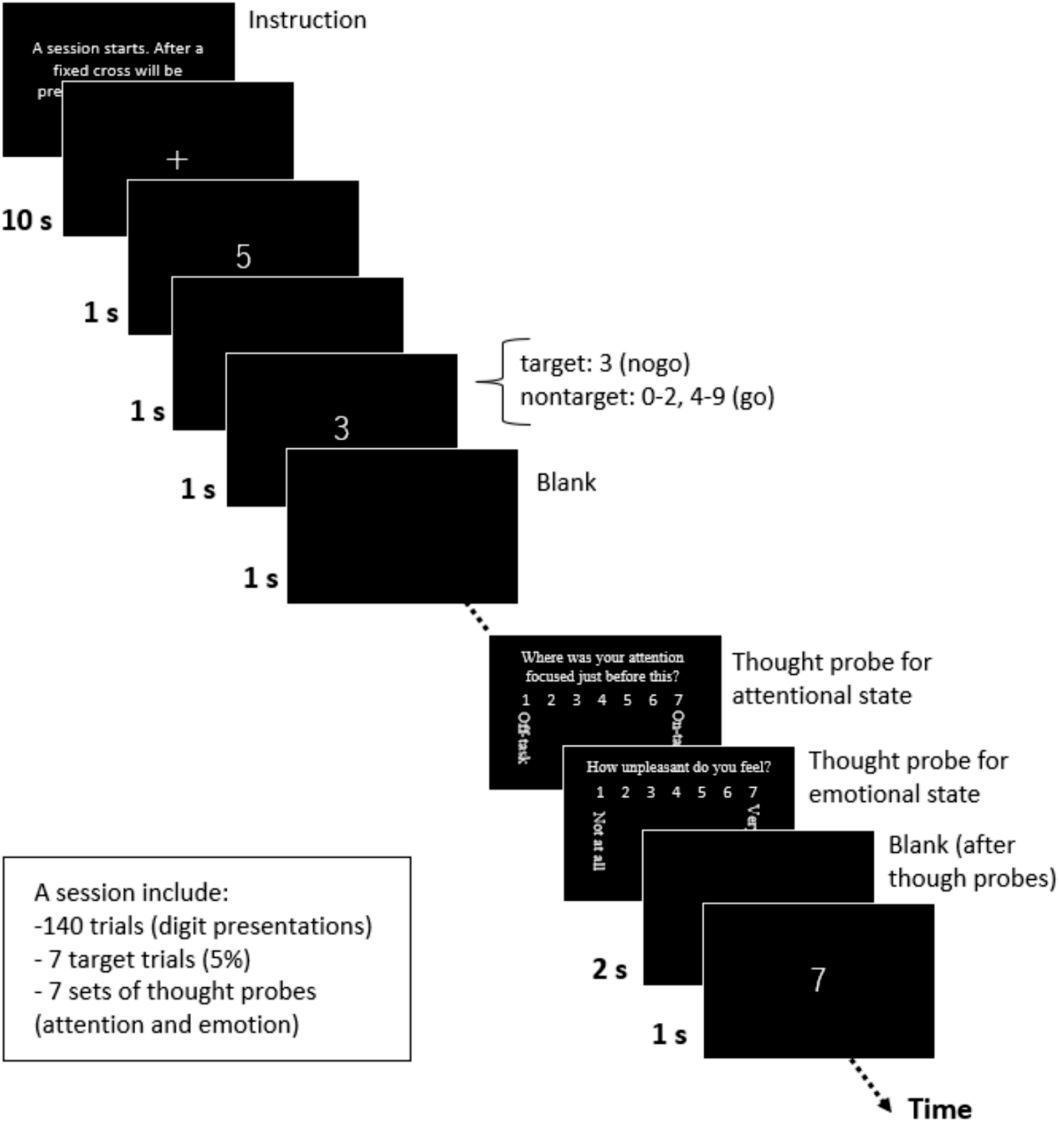
A SART session. A Sustained Attention to Response Tasks (SART) session was completed as a distraction task.

In the procedure (Figure 2), participants were initially given the following instructions through a monitor: “A session starts. After a fixation cross will be presented for a while, the digits will be presented. Please press a key as soon as the digit appears. Please do not press a key if the digit is 3. Press a key when you are ready (to go on the next monitor).” Next, a fixation cross was displayed at the center of the monitor as a baseline for 10 s. Following this, a series of digits (0–9) was presented serially and randomly at the center of the monitor every 2 s, with a target digit of 3 included in 5% of the trials, as noted by Christoff et al. (2009). This no-go rate was set according to previous studies (Christoff et al., 2009; Hu et al., 2012; and Marchetti et al., 2012). The session consisted of 140 trials (digit presentations), including seven target trials in the present study. In each trial, a white digit appeared on a black background for 1 s, followed by a blank screen for 1 s. Participants were instructed to press a key with their right index finger when a non-target digit appeared, but to withhold pressing when the target digit (3) was presented.

During the session, we presented a thought probe to assess the participants’ attentional state just before it appeared (Hu et al., 2012; Marchetti et al., 2012; McVay and Kane, 2009). Our thought probe included two types of questions related to participant’s attentional and emotional states. The first question was, “Where was your attention focused just before this?” Participants responded on a 7-point scale (1: *on-task*, 7: *off-task*) based on Christoff et al. (2009). We designed this attention scale to measure the degree of task-relatedness of thoughts, as defined by Stawarczyk et al. (2011), and explained it to participants as follows: “On-task” included complete focus on the task or thinking about nothing; “Task-related thoughts” involved appraisals of the current task (task-related interference), such as the consideration of task duration and personal performance; and “Off-task” included TUTs, such as noticing environment noises and physical sensations (external distractions), as well as thoughts like planning today’s dinner or recalling past unpleasant memories (mind-wandering). After the participants responded to this question by pressing a key, a second question appeared: “How unpleasant do you feel?” Participants responded on a 7-point scale (1 = *not at all*, 7 = *very*). The second question was based on a previous study on emotional distraction (Van Dillen et al., 2009).

After the participants responded to the second question, a blank screen was presented for 2 s. The series of digits were then resumed. The participants completed one SART session that included seven presentations of thought probes. Each thought probe appeared at a pseudo-random interval, with a minimum spacing of 10 s between probes. On average, it took approximately 359 s to complete the SART. All task stimuli were created and participants’ responses were recorded using E-Prime software (version 2.0; Psychology Software Tools, Sharpsburg, PA, USA).

Additionally, a split-half reliability of the SART was examined using response times (RTs) and coefficient of variations (CVs; SD-RT/mean RT) for nontargets, which are indices used in later behavioral analysis. The split-half reliabilities for the RTs and CVs were *r_sb_* = 0.91 and 0.55, respectively, calculated using the averaged values of the RTs and CVs for the first three (R1, R2, and R3) and later four (R4, R5, R6, and R7) rounds during the SART.

### 2.6 NIRS data acquisition and pre-processing for the SART

#### 2.6.1 NIRS recording

We used a Spectratech OEG-16H (Spectratech Inc.), which operates at two near-infrared wavelengths (770 and 840 nm). Six emitter and six detector optodes were positioned 3 cm apart in the PFC (15 × 3 cm), resulting in 16 channels. Changes in oxygenated hemoglobin (oxyHb), deoxygenated hemoglobin (deoxyHb), and total hemoglobin (Hb) concentrations were recorded for each of the 16 channels. The sampling interval was 81.92 ms. The probe holder was placed on Fpz, following the international 10–20 system. The measurement areas covered the superior frontal gyrus (Fp1 and Fp2), inferior frontal gyrus (F7 and F8), and inferior part of the DLPFC (F3 and F4) (Okamoto et al., 2004). We assessed NIRS data during SART for the research aim of the present study but recorded NIRS data during both the emotion induction task and SART for other research aims.

#### 2.6.2 Pre-processing of NIRS data for the SART

We focused on examining the changes in oxyHb concentration. Prior to analysis, raw oxyHb data were band-pass filtered using a high-pass filter set at 8.3 mHz to remove long-time drift artifacts and a low-pass filter set at 150 mHz to eliminate respiration, heartbeat, and other high-frequency noise artifacts (Pinti et al., 2015). Artifacts from changes in skin blood flow in the superficial head layers were removed using the hemodynamic modality separation method (Yamada et al., 2012).

Data were then converted to z-scores for each channel by calculating the mean (M) and standard deviation (SD) of oxyHb changes during the last 5 s (61 samples) before digit presentation onset during the SART, which served as the baseline of the SART. Consequently, baseline M ± SD values were converted to z-scores of 0 and 1 for each channel (Matsuda and Hiraki, 2006), and the z-scores during the tasks represent differences in the oxyHb changes from the baseline. The differences between sampled data were calculated for artifact rejection. Participant’s data with 1 or more difference value exceeding ± 10 SD were excluded, resulting in the removal of three participants.

### 2.7 Behavioral and NIRS data analysis

#### 2.7.1 Descriptive data for the Cutaneous9 and group division

We initially examined the Ms. ± SDs for the total Cutaneous9 scale score and individual item scores. Sex differences in the Cutaneous9 scale score were analyzed using Student’s *t*-tests. Next, the participants were divided into higher and lower Cutaneous9-score groups based on the M Cutaneous9 score. Student’s *t*-test was then used to examine scale score differences between the two groups. Additionally, Student’s *t*-test was applied to each Cutaneous9 item score to further characterize group differences.

#### 2.7.2 Emotional changes during the induction and distraction tasks

To examine emotional changes resulting from the emotion induction task, we calculated the Ms. ± SDs for the pre- and post-unpleasant emotion item scores. Sex differences in these item scores were also analyzed. We hypothesized that the emotion induction task would increase negative emotions in both the higher and lower Cutaneous9-score groups. This hypothesis was tested using a two-way mixed analysis of variance (ANOVA), with group (higher and lower Cutaneous9-score groups) and task period (pre-induction and post-induction) as factors for pre- and post-unpleasant emotion item scores.

Next, we examined emotional changes across the emotion induction and distraction tasks. Initially, we calculated the Ms ± SDs at pre-induction, post-induction, and post-distraction. Sex differences in these scores were examined using Student’s *t*-tests. We hypothesized that the emotion induction task would increase negative emotions and decrease positive emotions in both the higher and lower Cutaneous9-score groups, whereas the distraction task would specifically decrease negative emotions in the lower group as a distraction effect. To test these hypotheses, we conducted a two-way mixed ANOVA with group (higher and lower Cutaneous9-score groups) and task period (pre-induction, post-induction, and post-distraction) as factors affecting PANAS NA and PA scores, followed by Bonferroni post-hoc tests.

#### 2.7.3 Comparison of behavioral responses and PFC activities during distraction between the groups

Attention scores, rated from 1 (*on-task*) to 7 (*off-task*) during SART, were inverted to a scale from 1 (*off-task*) to 7 (*on-task*), indicating that higher scores reflect a greater level of attention. Emotion scores, rated from 1 (*not at all*) to 7 (*very unpleasant*), were used without adjustment so that higher scores reflected a greater degree of an unpleasant emotional state. These ratings were collected seven times during the SART, and we calculated the average of these scores for each participant separately for attention and emotion, defining them as grand-averaged attention and emotion scores. We hypothesized that the higher Cutaneous9-score group would demonstrate a lower attentional state and a higher unpleasant emotional state. To examine this, a one-way multivariate analysis of variance (MANOVA) was conducted, with the group as the independent variable and grand-averaged attention and emotion scores as the dependent variables.

To examine SART performance, we used RTs and CVs for nontargets, as these values have been examined in relation to the frequency of mind-wandering or levels of attentional states (Cheyne et al., 2009; Stawarczyk et al., 2011). We calculated average RTs and CVs over the 10 s prior to each thought probe (which included five responses), following Christoff et al. (2009). Thought probes were presented seven times, and we calculated the grand-averaged RTs and CVs across these seven intervals. Faster RTs and greater RT variabilities are generally associated with subsequent errors and considered indicators of attentional lapses (deBettencourt et al., 2019; Robertson et al., 1997). We hypothesized that the higher Cutaneous9-score group would exhibit shorter RTs and greater CVs than the lower group and conducted a one-way MANOVA with group as the factor to examine these grand-averaged RTs and CVs as dependent variables.

To analyze prefrontal activity, we calculated the average z-scores of oxyHb changes over the 10 s prior to each thought probe for each channel, according to Christoff et al. (2009). The baseline was set as the oxyHb change over the last 5 s before digit presentation onset, as previously mentioned. We then computed the grand-averaged oxyHb changes (z-scores) across seven periods. We hypothesized that the higher Cutaneous9-score group would exhibit lower activation in the LPFC regions and higher activation in the MPFC regions than the lower group. To test this hypothesis, we conducted a one-way MANOVA with the group as the factor and grand-averaged oxyHb changes (z-scores) across the 16 channels as dependent variables.

#### 2.7.4 Comparison of changes in behavioral responses and PFC activities during distraction between the groups

We hypothesized that the higher Cutaneous9-score group would show reduced attentional focus on the ongoing task and would exhibit a greater intensity of unpleasant emotions than the lower group. To examine how attentional and emotional states changed during the SART for each group, we conducted a two-way mixed-design repeated-measures ANOVA with group (higher or lower) and period (1st to 7th) as independent factors of attention and emotion scores. A Bonferroni post-hoc test was performed, as appropriate.

Additionally, we hypothesized that the higher Cutaneous9-score group would show decreased RTs and increased CVs during SART compared with the lower group, based on the assumption of decreased attentional focus on the task. To examine this, we conducted a two-way mixed-design repeated-measures ANOVA with group (higher or lower) and period (1st to 7th) as independent factors on the values of RTs and CVs.

Furthermore, we hypothesized that oxyHb changes (z-scores) in the higher Cutaneous9-score group would be smaller in the LPFC regions and greater in the MPFC regions during distraction when compared with the lower group, as we assumed decreased attentional focus and increased TUTs and cognitive processing in the higher Cutanous9 group. To investigate this, we conducted a two-way mixed-design repeated-measures ANOVA using group (higher or lower) and period (1st to 7th) as factors on the oxyHb changes for each channel independently. Bonferroni post-hoc tests were performed as required.

All analyses were performed using Microsoft Excel (Microsoft Corporation, WA, USA), SPSS Statistics (version 27.0; IBM Corp., Armonk, NY, USA), and MATLAB R2018b (MathWorks, Natick, MA, USA).

## 3 Results

### 3.1 Descriptive data of the Cutaneous9 score and group division

The Ms. ± SDs for the total Cutaneous9 score and individual item scores are shown in Table 1. There were no significant sex differences in the total Cutaneous9 score. The highest item score was “pain” (2.35 ± 4.08), while the lowest was “tingling” (0.18 ± 0.69). Of the 57 participants, 13 (22.8%) scored zero on the Cutaneous9 total scale, indicating that 77.2% of the participants reported experiencing at least some level of these symptoms.

**Table 1:** Means and standard deviations for the Cutaneous9 scale and item scores.

Scale (possible range score)	Total group	Higher group	Lower group	*t* values	*p*
	Mean (standard deviation)	Mean (standard deviation)			
Cutaneous9 (0–1782)	10.81 (12.09)	23.52 (11.01)	3.39 (3.36)	8.16	< 0.001***
Burning	1.37 (3.19)	2.90 (4.58)	0.47 (1.44)	2.37	0.027*
Crawling sensation	1.30 (3.17)	3.10 (4.64)	0.25 (0.84)	2.79	0.011*
Tingling	0.18 (0.69)	0.38 (1.02)	0.06 (0.33)	1.41	0.171
Pricking or pins and needles	2.11 (4.23)	4.81 (5.98)	0.53 (1.28)	3.23	0.004**
Pain	2.35 (4.08)	4.90 (5.53)	0.86 (1.73)	3.26	0.004**
Tender to touch	0.19 (0.83)	0.19 (0.87)	0.19 (0.82)	0.02	0.986
Numbness	0.21 (1.11)	0.29 (1.31)	0.17 (1.00)	0.39	0.701
Moderate to severe itching	2.30 (4.97)	5.05 (7.07)	0.69 (1.95)	2.76	0.011*
Easy bruising	0.81 (2.47)	1.90 (3.77)	0.17 (0.74)	2.09	0.049*

*p < 0.05, **p < 0.01, **p < 0.001.

Participants were divided into higher and lower groups based on a Cutaneous9 mean score of 10.81 (see Supplementary Figure 1). The higher group consisted of 21 participants (7 males and 14 females), while the lower group included 36 (15 males and 21 females). Student’s *t-*test confirmed that the higher group had significantly higher Cutaneous9 scores than the lower group (*t* (22.19) = 8.16, *p* < 0.001, *d* = 2.81; Table 1). For individual Cutaneous9 item scores, the higher group scored significantly higher on “burning” (*t* (22.34) = 2.37, *p* = 0.027, *d* = 0.81), “crawling sensation” (*t* (20.77) = 2.79, *p* = 0.011, *d* = 0.98), “pricking or pins and needles” (*t* (21.06) = 3.23, *p* = 0.004, *d* = 1.14), “pain” (*t* (22.30) = 3.26, *p* = 0.004, *d* = 1.12), “moderate to severe itching” (*t* (21.80) = 2.76, *p* = 0.011, *d* = 0.96), and “easy bruising” (*t* (20.90) = 2.09, *p* = 0.049, *d* = 0.74) based on Student *t*-tests.

### 3.2 Comparisons of emotional changes during the emotion induction and distraction tasks between the groups

The Ms ± SDs for the pre- and post-unpleasant emotion scores during the emotion induction task are shown in Table 2. No significant sex differences were found for any of the scores. A two-way mixed ANOVA with factors for group (higher and lower) and task period (pre-induction and post-induction) on unpleasant emotion scores revealed a main effect for the task period (*F*(1, 55) = 102.43, *p* < 0.001, *η_p_^2^* = 0.65). Post-scores were higher than pre-scores (*p* < 0.001, *d* = 1.94; Bonferroni post-hoc test), which is consistent with our previous studies using the same emotion induction paradigm (*d* = 1.83 in Ozawa et al., 2020; *d* = 1.93 in Ozawa, 2021; *d* = 1.59 in Ozawa et al., 2022).

**Table 2:** Means and standard deviations for unpleasant emotion and PANAS NA and PA scores.

Group	*N* (male)	Unpleasant emotion		PANAS NA			PANAS PA			PANAS dif values	
		Pre-induction	Post-induction	Pre-induction	Post-induction	Post-distraction	Pre-induction	Post-induction	Post-distraction	NA dif values	PA dif values
Total group	57 (22)	1.33 (0.58)	3.63 (1.58)	12.04 (5.28)	18.14 (5.99)	14.30 (7.15)	16.26 (5.99)	13.05 (5.05)	13.40 (5.31)	−3.84 (6.58)	0.35 (5.38)
Higher group	21 (7)	1.43 (0.68)	3.62 (1.56)	13.14 (6.53)	17.48 (6.59)	16.33 (9.32)	15.76 (4.96)	13.71 (5.07)	13.14 (4.07)	−1.14 (6.79)	−0.57 (4.86)
Lower group	36 (15)	1.28 (0.51)	3.64 (1.61)	11.39 (4.36)	18.53 (5.66)	13.11 (5.31)	16.56 (6.57)	12.67 (5.07)	13.56 (5.96)	−5.42 (5.99)	0.89 (5.65)

PANAS, Positive and Negative Affect Schedule; NA, negative affect; PA, positive affect; dif, difference. The PANAS NA or PA difference values were subtracted from the post-induction and post-distraction scores.

**Figure 3:**
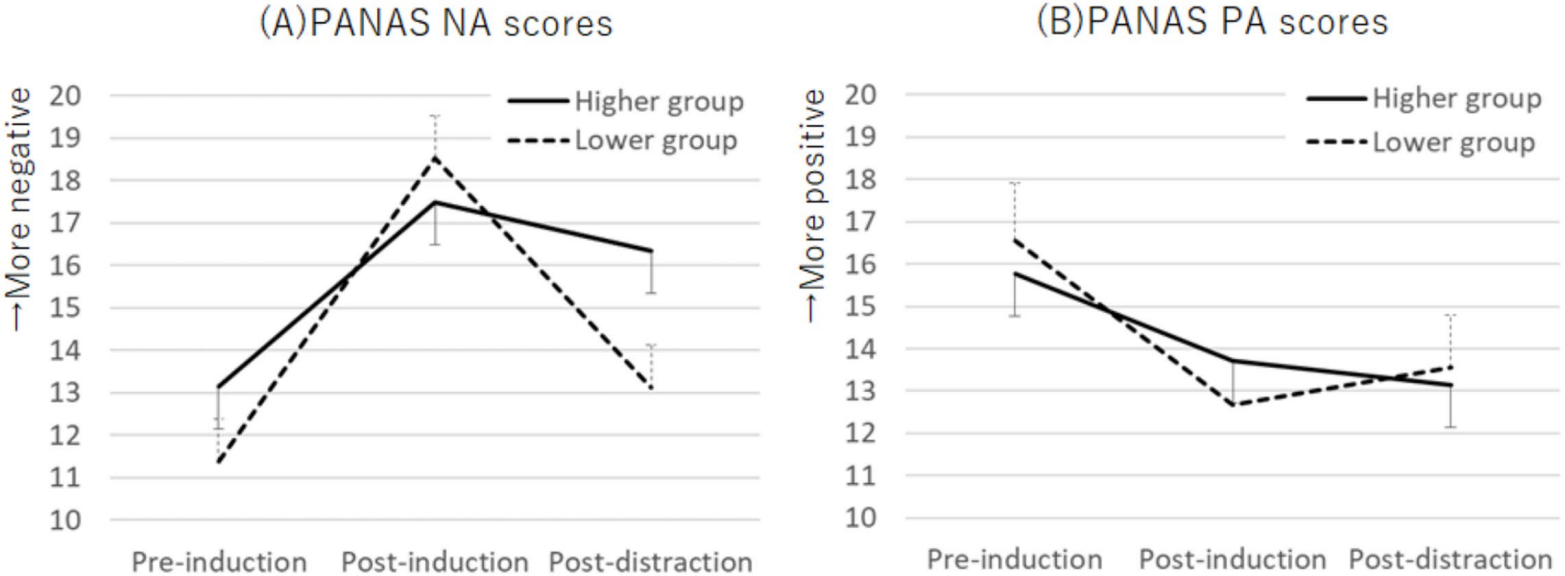
Plots of PANAS NA scores **(A)** and PANAS PA scores **(B)** across the emotion induction and distraction tasks. An interaction effect was observed for PANAS NA scores, whereas PANAS PA scores showed a main effect during the task period. Error bars represent standard error. PANAS, Positive and Negative Affect Schedule; PA, positive affect; NA, negative affect.

The Ms. ± SDs for the PANAS NA and PA scores at pre-induction, post-induction, and post-distraction are shown in Table 2. The Cronbach’s alphas for the PANAS NA scores at pre-induction, post-induction, and post-distraction were 0.86, 0.71, and 0.87, respectively, while those for the PANAS PA scores were 0.79, 0.76, and 0.81, respectively. No significant sex differences were found for any of the scores. A two-way mixed ANOVA with factors for group (higher and lower) and task period (pre-induction, post-induction, and post-distraction) on the PANAS NA scores showed an interaction effect (*F* (2, 110) = 3.96, *p* = 0.022, *η_p_^2^* = 0.07; Figure 3A). In the higher group, both post-induction and post-distraction scores were higher than the pre-induction scores (*p* = 0.001, *d* = 0.66; *p* < 0.001, *d* = 0.40). In the lower group, post-induction scores were higher than pre-induction scores (*p* < 0.001, *d* = 1.41), whereas post-distraction scores were lower than post-induction scores (*p* < 0.001, *d* = 0.35). For PANAS PA scores, a two-way mixed ANOVA indicated a main effect for task period (*F* (2, 110) = 11.68, *p* < 0.001, *η_p_^2^* = 0.18). Both post-induction and post-distraction scores were lower than the pre-induction scores (*p* < 0.001, *d* = 0.58; *p* < 0.001, *d* = 0.51; Bonferroni post-hoc test; Figure 3B).

### 3.3 Comparisons of the behavioral responses and PFC activities during distraction between the groups

#### 3.3.1 Grand-averaged attention and emotion scores

The M ± SD values for the grand-averaged attention and emotion scores in each group are shown in Table 3. A one-way MANOVA indicated no significant differences between the groups in either score.

**Table 3:** Means and standard deviations for the SART responses and their differences between the higher and lower Cutaneous9-score groups.

Indices	Mean (standard deviation)		*F* values	*p*
	Higher group	Lower group		
Grand-averaged self-report scores				
Attention scores	5.01 (1.29)	5.49 (0.99)	2.50	0.120
Unpleasant emotion scores	2.04 (0.98)	1.88 (0.83)	0.43	0.516
Grand-averaged SART responses				
RTs for nontargets	342.31 (45.26)	358.42 (54.36)	1.31	0.257
CVs for nontargets	0.13 (0.05)	0.13 (0.03)	0.08	0.779
Grand-averaged oxyHb changes (z-scores)				
ch1	5.13 (6.88)	4.01 (9.42)	0.03	0.855
ch2	12.34 (16.08)	5.33 (15.59)	2.80	0.101
ch3	8.01 (12.06)	4.43 (13.32)	0.37	0.548
ch4	6.09 (11.15)	7.16 (17.00)	0.16	0.696
ch5	4.26 (15.87)	5.41 (11.75)	0.00	0.954
ch6	5.64 (14.57)	4.95 (10.97)	0.01	0.910
ch7	4.11 (11.00)	5.36 (13.59)	0.16	0.693
ch8	15.23 (22.64)	6.13 (13.37)	5.13	0.028*
ch9	2.46 (8.05)	3.58 (7.57)	0.05	0.817
ch10	9.66 (11.43)	3.76 (8.41)	4.97	0.031*
ch11	16.73 (19.57)	3.80 (18.75)	5.45	0.024*
ch12	7.86 (16.87)	6.63 (11.84)	0.03	0.856
ch13	6.24 (8.98)	2.43 (10.08)	2.36	0.131
ch14	7.37 (13.00)	4.76 (9.52)	1.56	0.219
ch15	5.73 (9.77)	6.13 (7.61)	0.10	0.759
ch16	6.21 (13.28)	5.48 (14.14)	0.10	0.759

SART, Sustained Attention to Response Tasks; RTs, response times; CV, coefficient of variation; oxyHb, oxygenated hemoglobin; Ch, channel. *p < 0.05.

#### 3.3.2 Grand-averaged RTs and CVs for nontargets

Table 3 also shows the M ± SD values for the grand-averaged RTs and CVs for nontargets in each group. A one-way MANOVA revealed no significant differences between the groups in any of these scores.

Additionally, Supplementary Table 1 shows correlations among grand-averaged RTs and CVs for nontargets, commission error (CE) rates for targets, and attention scores during SART.

#### 3.3.3 Grand-averaged OxyHb changes

**Figure 4:**
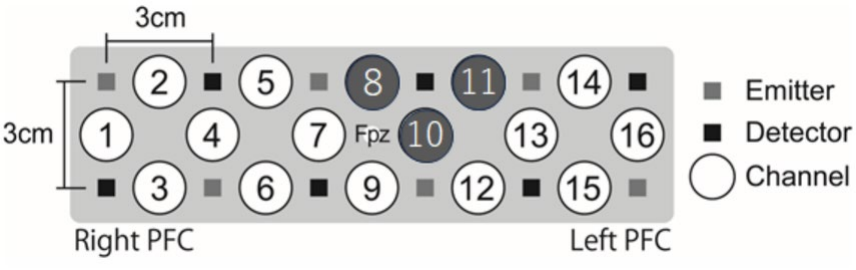
NIRS settings and locations of ch8, 10, and 12. The higher Cutaneous9-score group showed a greater increase in oxyHb changes than the lower score group during SART at ch8, 10, and 12. NIRS, near-infrared spectroscopy; PFC, prefrontal cortex.

The M ± SD values for grand-averaged oxyHb changes (z-scores) in each group are also presented in Table 3. The higher group showed significantly greater changes in oxyHb increases than the lower group in channel (ch) 8, 10, and 11 (Figure 4; ch8: *F* (1, 45) = 5.13, *p* = 0.028, *η_p_^2^* = 0.10; ch10: *F* (1, 45) = 4.97, *p* = 0.031, *η_p_^2^* = 0.10; ch11: *F* (1, 45) = 5.45, *p* = 0.024, *η_p_^2^* = 0.11; one-way MANOVA). No other significant differences were observed between the groups.

### 3.4 Comparison of changes in behavioral responses and PFC activities during distraction between the groups

#### 3.4.1 Changes in the attention and emotion scores

**Figure 5:**
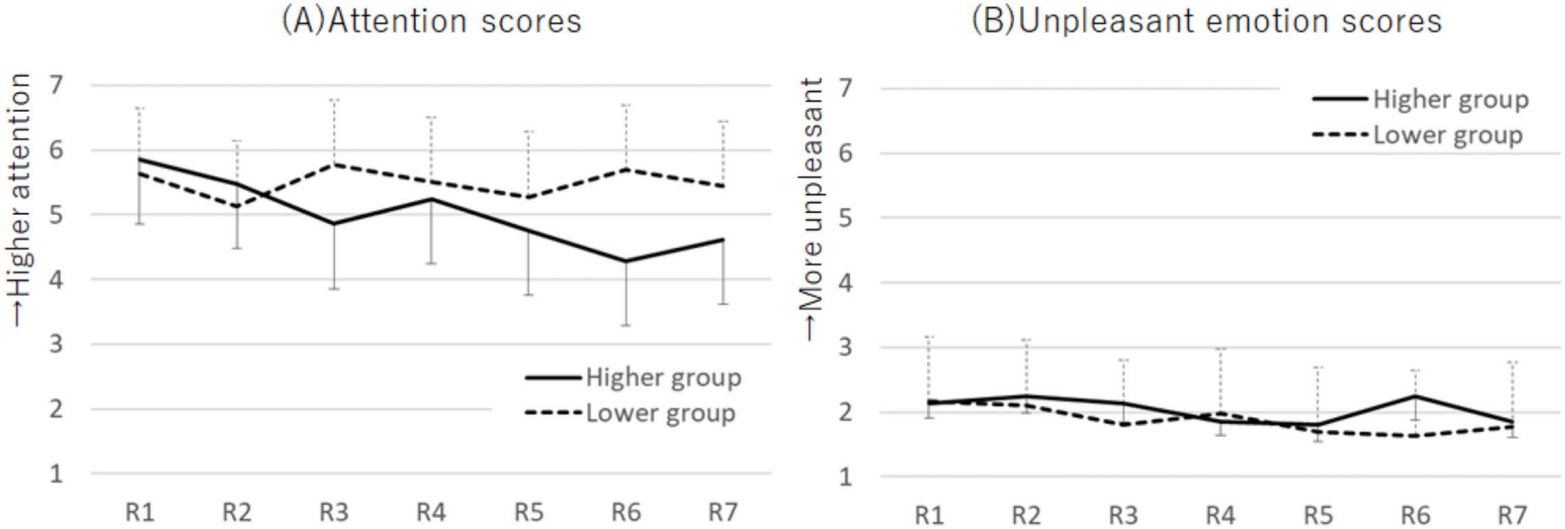
Plots of the attention scores **(A)** and unpleasant emotion scores **(B)** across each round during the SART for the higher and lower Cutaneous9-score groups. The attention scores exhibited an interaction effect, whereas the unpleasant emotion scores showed no effect. Error bars represent standard error.

The M ± SD values of the attention and emotion scores for each period by group are shown in Supplementary Table 2. For the attention scores, a two-way mixed ANOVA with factors of group (higher and lower) and period (1st to 7th) revealed an interaction effect (*F* (5.12, 281.55) = 3.18, *p* = 0.008, *η_p_^2^* = 0.06; Figure 5A). The Bonferroni post-hoc test showed that, in the higher group, attention scores in round 6 were lower than those in round 1 (*p* = 0.021, *d* = 0.92). Additionally, the higher group had lower attention scores than the lower group in rounds 3 (*p* = 0.034, *d* = 0.60) and 6 (*p* = 0.004, *d* = 0.84).

Regarding emotion scores, no significant effects were found using the two-way mixed ANOVA (Figure 5B). Therefore, to further examine changes in unpleasant emotion scores across emotion induction and distraction tasks, we performed a two-way mixed ANOVA with factors for group (higher and lower) and task period (pre-induction, post-induction, and R1) on unpleasant emotion scores. It revealed a main effect for the task period (*F* (2, 110) = 73.92, *p* < 0.001, *η_p_^2^* = 0.57). Post-induction scores were higher than pre-induction scores (*p* < 0.001, *d* = 1.63; Bonferroni post-hoc test). R1 scores were lower than post-induction scores (*p* < 0.001, *d* = 1.33) and higher than pre-induction scores (*p* < 0.001, *d* = 0.62). Similarly, a two-way mixed ANOVA with factors for group (higher and lower) and task period (pre-induction, post-induction, and R7) on unpleasant emotion scores revealed a main effect for the task period (*F* (2, 110) = 67.73, *p* < 0.001, *η_p_^2^* = 0.55). Post-induction scores were higher than pre-induction scores (*p* < 0.001, *d* = 1.63). R7 scores were lower than post-induction scores (*p* < 0.001, *d* = 0.41) and higher than pre-induction scores (*p* = 0.001, *d* = 1.00).

#### 3.4.2 Changes in the RTs and CVs for nontargets

**Figure 6:**
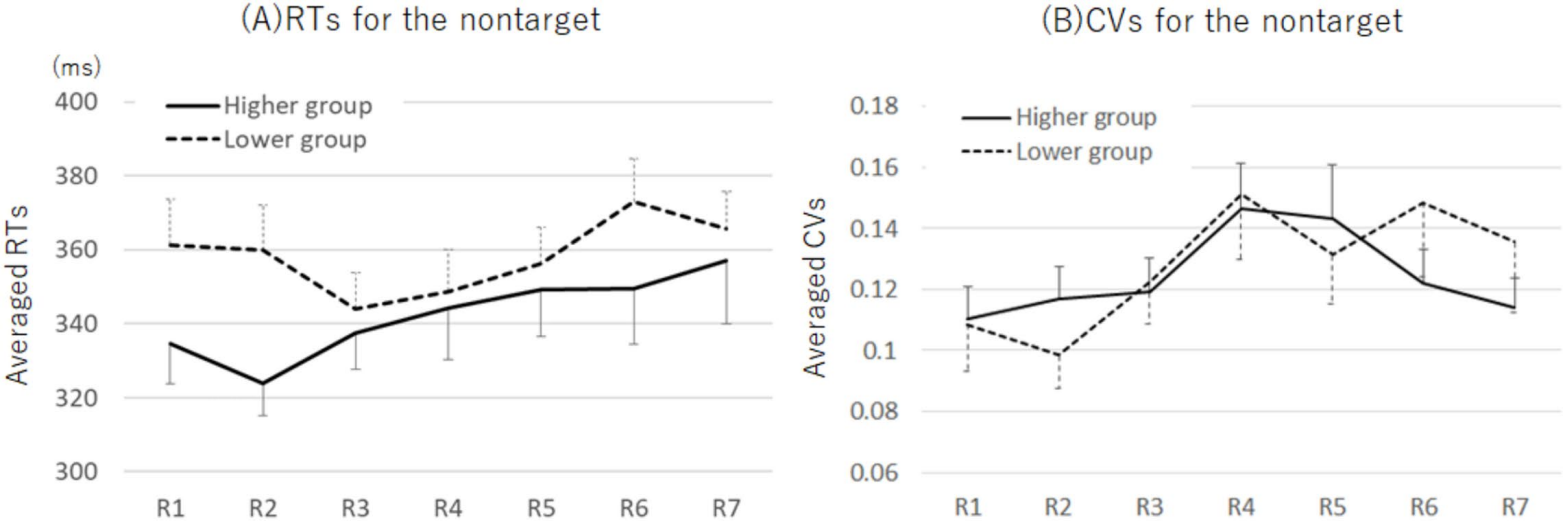
Plots of the RTs **(A)** and CVs **(B)** at each round during the SART for the higher and lower Cutaneous9-score groups. No significant differences were observed across any conditions in the analysis of the RTs of the nontarget. The CVs were higher at R4 than at R2. Error bars represent standard errors. RT, response time; SART, Sustained Attention to Response Tasks; CV, coefficient of variation.

The M ± SD values of the RTs and CVs for nontargets across each period by group are presented in Supplementary Table 2. A two-way mixed ANOVA examining the effects of group (higher and lower) and period (1st–7th) on the RTs revealed no effect (Figure 6A). The analysis of CVs showed a main effect of period (two-way mixed ANOVA; *F* (6, 330) = 2.34, *p* = 0.032, *η_p_^2^* = 0.04; Figure 6B). Bonferroni post-hoc test demonstrated that the CVs in the 4th round were higher than those in the 2nd round (*p* = 0.040, *d* = 0.27).

#### 3.4.3 Changes in the OxyHb changes

**Figure 7:**
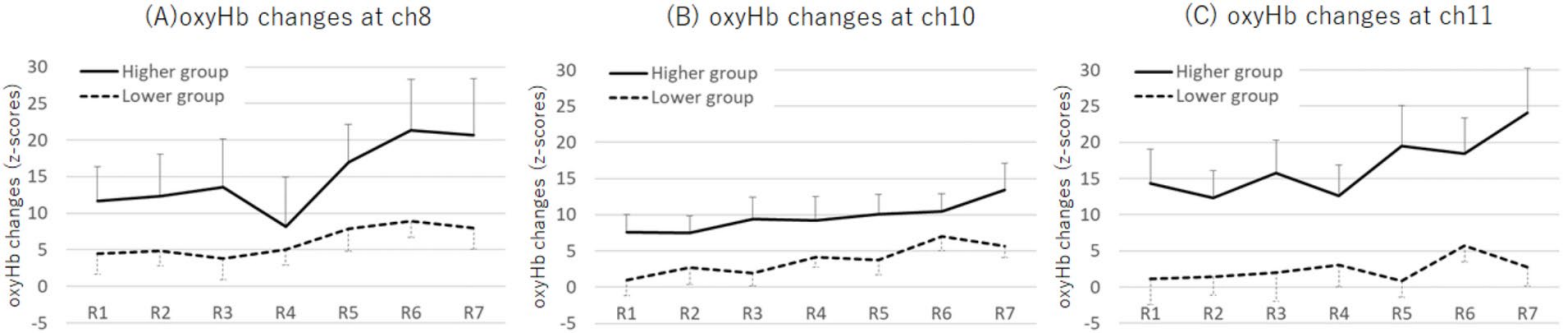
Plots of oxyHb changes (z-scores) at ch8 **(A)**, ch10 **(B)**, and ch11 **(C)** across each round during the SART for the higher and lower Cutaneous9-score groups. An interaction effect between group and round was observed at ch11, where the higher group exhibited greater oxyHb changes in the 7th round compared with that in the 2nd, 3rd, and 4th rounds. Error bars represent standard error. Ch, channel; SART, Sustained Attention to Response Tasks; oxyHb, oxygenated hemoglobin.

The M ± SD values of the oxyHb changes (z-scores) for each period by group are shown in Supplementary Table 2. A two-way mixed ANOVA, with factors of group (higher and lower) and period (1st to 7th), revealed an interaction effect at ch11 (*F* (4.06, 202.73) = 2.90, *p* = 0.022, *η_p_^2^* = 0.55; Figure 7). The Bonferroni post-hoc test demonstrated that the higher group exhibited greater increases in oxyHb changes across all rounds than the lower group (1st: *p* = 0.001, *d* = 0.63; 2nd: *p* = 0.015, *d* = 0.71; 3rd: *p* = 0.002, *d* = 0.64; 4th: *p* = 0.020, *d* = 0.53; 5th: *p* = 0.001, *d* = 0.95; 6th: *p* = 0.011, *d* = 0.77; 7th: *p* = 0.001, *d* = 0.77). Additionally, for both groups, the increase in oxyHb changes in the 6th round was greater than that in the 2nd round (higher: *p* = 0.005, *d* = 0.32; lower: *p* = 0.012, *d* = 0.31), and the increase during the 5th round was greater than that in the 1st round (higher: *p* < 0.001, *d* = 0.23; lower: *p* = 0.050, *d* = 0.02). Notably, for the higher group, the increase in oxyHb changes during the 7th round was greater than those during the 2nd, 3rd, and 4th rounds (2nd: *p* = 0.001, *d* = 0.53; 3rd: *p* < 0.001, *d* = 0.36; 4th: *p* = 0.004, *d* = 0.50). In contrast, for the lower group, the increase in oxyHb changes during the 6th round was greater than in the 1st and 3rd rounds (1st: *p* = 0.044, *d* = 0.27; 3rd: *p* = 0.047, *d* = 0.20). No other significant differences were observed between the groups (see Supplementary Figure 2 for all channels).

## 4 Discussion

This study examined whether non-clinical healthy students with higher versus lower levels of cutaneous symptoms would show differences in distraction effects to unpleasant emotions. Consistent with the findings of Gupta and Gupta (2004), who highlighted the prevalence of cutaneous sensory symptom experiences in non-clinical populations, we observed that a notable number of healthy students without any psychiatric, neurological, or dermatological diseases reported experiencing such cutaneous symptoms.

We first investigated whether participants with higher and lower cutaneous symptoms showed different degrees of emotional change in response to emotional induction and distraction tasks. Following the recall of stressful interpersonal events, both groups experienced a significant increase in negative emotions, as measured by an unpleasant emotion item and the PANAS NA, along with a decrease in positive emotions, as assessed by the PANAS PA. This confirmed that emotion induction was effective, regardless of the level of cutaneous symptoms. However, the distraction task (SART) successfully reduced unpleasant emotions, as measured by the PANAS NA, only in the group with lower cutaneous symptoms. In the higher-symptom group, negative emotions remained elevated even after the distraction task. These results suggest that individuals with more severe cutaneous symptoms have more difficulty in reducing unpleasant emotions through distraction than those with less severe cutaneous symptoms.

However, unpleasant emotion measured using the unpleasant emotion item during the SART demonstrated no reduction. We previously found that after recalling stressful daily interpersonal events, the unpleasant item was relatively strongly associated with anger among six emotions (surprise, fear, anger, disgust, sadness, and happiness; Ozawa, 2021), indicating its conceptual difference from the PANAS NA. We assume that the unpleasant emotion, involving anger, might be relatively quickly reduced after emotion induction, making it unlikely to attenuate more during distraction. This is based on the reduction in unpleasant emotions at R1, the beginning of the SART, compared to those at the post-induction period.

We assumed that the effects of distraction on unpleasant emotion would be related to the degree of attentional focus on the SART. Our analysis of changes in the attention scores indicated that the higher cutaneous symptom group showed lower attentional focus than the lower cutaneous symptom group during the 3rd and 6th periods. Additionally, only the higher symptom group exhibited a decrease in attentional focus during the 6th period compared with the 1st, suggesting a decline in focus approximately 2 min after the task began. These findings regarding the reduction in attentional focus in the higher group indicate a possible increase in various cognitive and emotional processing. Thus, our findings indicate that participants with more severe cutaneous symptoms have greater difficulty shifting their attention away from unpleasant emotions, particularly after stressful events. This tendency could predispose them to ruminating about unpleasant issues, potentially prolonging their negative mood states in their daily lives.

The behavioral attention index of the CVs increased during the SART, indicating a decrease in attentional focus among participants in both groups. Considering that only participants with higher cutaneous symptoms showed decreases in subjective reports for the attentional scores, the findings between subjective and behavioral attention index appear to be inconsistent. Regarding this, it is noted that subjective and behavioral markers of sustained attention may fluctuate independently because they are based on different attentional mechanisms (Chidharom et al., 2025). Subjective markers are associated with the self-monitoring process of attention compared with behavioral markers that represent a more automatic process of attention. As the level of the task difficulty and attentional demands might be low for our non-clinical participants, behavioral responses are likely to be automatized. However, it is noted that both mind-wandering thoughts and behavioral response time stability are associated with default-mode network activation, including the MPFC (Kucyi et al., 2023).

Our results supported our hypothesis regarding MPFC activation, showing that the higher group had greater increases in oxyHb changes in the MPFC regions than the lower group. Specifically, our results demonstrated these increases in the dorsal MPFC regions at ch8, 10, and 11, based on an analysis of grand-averaged oxyHb changes. Notably, at ch11, the higher symptom group exhibited particularly increased oxyHb changes toward the end of the task period (7th) compared with earlier periods (2nd, 3rd, and 4th). This suggests a possible increase in self-focused and off-task thoughts. However, contrary to our expectations, LPFC activation showed no significant differences between the groups, suggesting no substantial differences in the cognitive control of emotion. Considering these results, the reduced effectiveness of distraction in the higher symptom group appears to stem from a tendency toward absorption in off-task thoughts, rather than a failure in emotional inhibition.

In summary, this study revealed that many healthy Japanese undergraduate students experienced cutaneous sensory symptoms. Although these symptoms may not indicate serious health issues, they may reflect underlying mental stress. Such subtle signs of stress among healthy students may often go unrecognized; however, identifying these signals is essential as mental stress can be present with only faint indications. Furthermore, this study showed that students with higher cutaneous symptoms had more difficulty alleviating unpleasant emotions through distraction, suggesting a potential link between these stress indicators and overall well-being. To prevent such stress from progressing into more serious issues and improve well-being, it is crucial for clinical psychology to emphasize mental health support for non-clinical populations and provide strategies to enhance the quality of life in a stressful society.

This study had a several limitations. First, we focused on university students, given that many may experience underlying mental stress while still developing emotion regulation skills; therefore, examining emotion regulation and mental health is critical for this age group. However, it is also important to extend this focus to non-clinical populations across a broader age range. Second, the Cutaneous9 total scale showed a low Cronbach’s alpha value (0.45). However, the previous study by Gupta and Gupta (2004) did not report the Cronbach’s alpha values, possibly because of the relatively independent nature of each item’s score. In the present study, we primarily used the Cutaneous9 scale score to categorize the participants into two groups and did not perform extensive statistical analyses based on these scores, making it unlikely to have a significant impact on our findings. Third, the sample size became small, especially after dividing groups. It is mainly due to rare rates of individuals with cutaneous symptoms in non-clinical populations. However, as previously noted, an *a priori* power analysis using G*Power supported our main findings. It indicated that the total sample size provides 95% power to detect a medium-sized effect for a two-way mixed ANOVA interaction effect between group and task period (pre-induction, post-induction, and post-distraction) on PANAS scores, as well as for a two-way mixed ANOVA interaction effect between group and period (1st to 7th period) on oxyHb changes. Fourth, the 5% no-go rate, appearing only once in a round (i.e., approximately 20 trials in 40 s), is based on previous studies (Christoff et al., 2009; Hu et al., 2012). However, this rate might be relatively low in the present study. Therefore, we could not calculate the CE rates for 10 s, although we assessed those during the total duration (see Supplementary Table 1). Furthermore, although the low-frequency of the no-go condition is generally associated with higher-level task difficulty than the high-frequency condition as it makes individuals prone to respond, requiring higher-level attentional need for inhibition (Braver et al., 2001), the number of trials in this study was markedly lower than that in those previous studies, where task duration was approximately 1 h. Therefore, overall task difficuly or sustained attentional needs might become low. Fifth, the behavioral index of RTs and CVs during SART did not serve as an effective marker for detecting group differences in attention. This may be due to several reasons, including the low split-half reliability of CVs, the limited number of trials, and a small sample size within each group. Therefore, the behavioral results obtained from SART are inconclusive, and this issue remains to be addressed in future studies. Sixth, it remained unclear whether the responses of TUTs obtained from thought probes in this study represent mind-wandering, as the task duration is shorter than that in previous studies, as mentioned. We consider that these responses reflect the occurrence of various cognitive and emotional processing. Seventh, as previously mentioned, it is noted that off-task thoughts and mind-wandering can serve as a distraction from ongoing tasks, inducing positive mood, especially when off-task thoughts are perceived as interesting (Franklin et al., 2013). However, we considered that TUT occurrence during the task is associated with a failure of distraction because we examined distraction effects from a priori induced unpleasant emotions rather than from the ongoing SART. Furthermore, it has been reported that negative mood more frequently generates off-task thoughts and attentional lapses during tasks than positive mood (Smallwood et al., 2009). Eighth, we could not enroll a rest condition in our experimental design; therefore, we cannot eliminate the possibility that observed group differences in emotional changes can be associated with differences in time-based emotional recovery rather than the distraction task. In our previous experiments, which enrolled a rest condition (Ozawa et al., 2024; Ozawa, 2021), engaging in attention-demanding tasks was found to be more effective in decreasing unpleasant emotions than resting, with non-constant tapping specifically decreasing unpleasant TUTs compared with the rest condition (Ozawa et al., 2024). However, the present study focused on the group differences regarding cutaneous symptoms; therefore, aspects of differences in a time-based emotional recovery are important. This issue remains to be addressed in future studies. Nineth, although we theoretically anticipated a link between self-reported attentional state and MPFC activation, further correlation analyses did not reveal any significant association. This may be because MPFC activation is influenced by various factors beyond the mere presence of thoughts. Tenth, the present study targeted participants with cutaneous sensory symptoms, but no cutaneous or mental disorders, including emotional disorders. However, we cannot exclude the possibility that they could be diagnosed with such disorders based on participants’ self-reports. Finally, this study focused on cutaneous sensory sensations as an indicator of underlying mental stress in non-clinical healthy students, revealing their associations with the effectiveness of emotional distraction. Our findings suggest that cutaneous sensory symptoms are particularly sensitive to subtle, implicit mental stress. However, it is essential to investigate other symptoms associated with underlying mental stress. Moreover, providing guidance on effective stress-coping strategies is crucial for supporting the mental health and resilience of these populations.

## 5 Conclusion

The present study demonstrated that many healthy university students experience cutaneous sensory symptoms that may serve as indicators of underlying stress. We observed that, after recalling stressful interpersonal events in their daily lives, participants with higher levels of cutaneous symptoms had greater difficulty focusing on the SART distraction task and reducing unpleasant emotions than those with lower levels of cutaneous symptoms. Furthermore, the higher symptom group exhibited increased activation of the MPFC during the distraction task, whereas no significant differences were found in LPFC activation. This suggests that the reduced effectiveness of distraction in the higher symptom group may be associated with a tendency toward absorption in off-task thoughts, which is characterized by MPFC activation, rather than a failure in emotional inhibition, which is particularly associated with LPFC activation. Our findings suggest a potential link between stress indicators and emotion regulation. To avert serious health and mental problems and promote well-being, greater attention should be paid to the mental health of non-clinical populations, along with the development of strategies to improve their quality of life.

## Data Availability

The raw data supporting the conclusions of this article will be made available by the authors, without undue reservation.

## Footnotes

^1^An *a priori* power analysis using G*Power (with an alpha error probability = 0.05) indicated that the sample size provides 95% power to detect a medium-sized effect (ηp2 = 0.06) for a two-way mixed ANOVA interaction effect between group and task period (pre-induction, post-induction, and post-distraction) on PANAS scores, as well as for a two-way mixed ANOVA interaction effect between group and period (1st to 7th period) on oxyHb changes.

^2^The questions about participants’ experienced emotions (e.g., “Could you remember easily, or did you need effort?”; “What kind of emotions did you experience?”) were intended to reduce emotional inhibition, which is known to negatively impact mental and physical health (Larson and Chastain, 1990). In contrast, self-disclosure or expression of emotions has been shown to promote positive outcomes by facilitating emotional release, or “catharsis,” and by enabling cognitive reconstructing of events through “cognitive reappraisal” (Pennebaker and Oheeron, 1984; Gross, 1998). Additionally, participants were asked about enjoyable daily life events at the end, aimed at redirecting their focus to more pleasant memories, a process known as “emotional distraction” (Nolenhoeksema and Morrow, 1993).
