# Supplementary material for: Cutaneous sensory symptoms and emotional regulation in non-clinical healthy students: a near-infrared spectroscopy study
Source: Front Psychol. 2025 Sep 22;16:1619280. doi: 10.3389/fpsyg.2025.1619280 (PMC12498142; doi:10.3389/fpsyg.2025.1619280)
Supplement: Supplementary file 1 [file Table_1.docx]

Supplementary Material


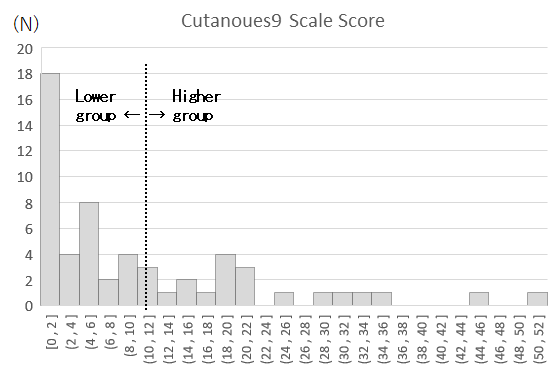


**Supplementary Figure 1.** Number of participants according to the Cutaneous9 scale score. The participants’ mean score was 10.81.

**Supplementary Table 1. Pearson’s Correlations between grand-averaged RTs and CVs for nontargets, CE rates for targets, and grand-averaged attention scores during SART**

|  |  | Mean (standard deviation) | 1 | 2 | 3 | 4 |
| --- | --- | --- | --- | --- | --- | --- |
| 1. Grand-averaged RTs for nontargets | | 352.49 (51.38) | - | - | - | - |
| 2. Grand-averaged CVs for nontargets | | .13 (.04) | .38** | - | - | - |
| 3. CE rates for targets | | .27 (.20) | -.39* | .24 | - | - |
| 4. Grand-averaged attention scores | | 5.32 (1.13) | -.03 | -.30* | -.30* | - |
| *Note*: SART: Sustained Attention to Response Tasks, RTs: response times, CVs: coefficient of variations, CEs: commission errors. The CE rates were calculated by dividing the number of no-go errors by the total number of no-go targets during the total duration of the SART. **p* < .05 and ***p* < .01. | | | | | | |

**Supplementary Table 2. Pearson’s Correlations between the behavioral responses and the oxyHb changes (z-scores) in the total study population**

|  |  | 1st | 2nd | 3rd | 4th | 5th | 6th | 7th |
| --- | --- | --- | --- | --- | --- | --- | --- | --- |
| Attention scores | | |  |  |  |  |  |  |
|  | Higher | 5.86 (0.64) | 5.48 (1.44) | 4.86 (1.68) | 5.24 (1.90) | 4.76 (1.81) | 4.29 (2.08) | 4.62 (2.13) |
|  | Lower | 5.64 (1.40) | 5.14 (1.76) | 5.78 (1.46) | 5.50 (1.72) | 5.28 (1.52) | 5.69 (1.41) | 5.44 (1.56) |
| Unpleasant emotion scores | | |  |  |  |  |  |  |
|  | Higher | 2.14 (1.11) | 2.24 (1.18) | 2.14 (1.35) | 1.86 (1.01) | 1.81 (1.21) | 2.24 (1.64) | 1.86 (1.11) |
|  | Lower | 2.17 (1.28) | 2.11 (1.41) | 1.81 (1.09) | 1.97 (1.28) | 1.69 (0.92) | 1.64 (0.90) | 1.78 (1.11) |
| RTs for nontargets | | |  |  |  |  |  |  |
|  | Higher | 334.67 (49.60) | 323.90 (40.34) | 337.43 (44.79) | 344.38 (64.17) | 349.24 (57.63) | 349.48 (68.70) | 357.10 (78.64) |
|  | Lower | 361.31 (74.59) | 359.92 (72.56) | 344.03 (59.52) | 348.81 (67.49) | 356.22 (59.58) | 373.03 (69.10) | 365.64 (61.14) |
| CVs for nontargets | | |  |  |  |  |  |  |
|  | Higher | .11 (.07) | .10 (.05) | .12 (.06) | .15 (.10) | .13 (.07) | .15 (.11) | .14 (.11) |
|  | Lower | .11 (.06) | .12 (.06) | .12 (.06) | .15 (.09) | .15 (.11) | .12 (.07) | .11 (.06) |
| OxyHb changes at ch1 | | |  |  |  |  |  |  |
|  | Higher | 3.80 (7.62) | 2.61 (8.7) | 6.76 (8.98) | 2.94 (8.16) | 5.63 (7.94) | 5.61 (8.93) | 8.53 (10.08) |
|  | Lower | 2.67 (11.96) | 2.45 (11.18) | 2.79 (10.00) | 3.75 (11.78) | 4.96 (12.61) | 5.77 (10.06) | 5.70 (10.47) |
| OxyHb changes at ch2 | | |  |  |  |  |  |  |
|  | Higher | 12.85 (16.93) | 10.74 (18.12) | 11.87 (20.1) | 7.67 (10.69) | 15.54 (19.16) | 14.02 (20.66) | 10.67 (10.68) |
|  | Lower | 3.27 (14.01) | 3.78 (11.64) | 2.6 (18.53) | 3.00 (12.17) | 7.63 (15.16) | 9.75 (16.05) | 7.28 (18.50) |
| OxyHb changes at ch3 | | |  |  |  |  |  |  |
|  | Higher | 4.18 (13.72) | 4.5 (8.57) | 11.58 (19.47) | 4.48 (16.31) | 7.07 (13.76) | 9.80 (10.73) | 11.92 (19.56) |
|  | Lower | -1.14 (16.19) | 5.75 (17.56) | 2.45 (15.83) | 3.52 (14.48) | 2.39 (14.99) | 9.98 (19.77) | 6.77 (16.57) |
| OxyHb changes at ch4 | | |  |  |  |  |  |  |
|  | Higher | 5.65 (11.59) | 4.61 (8.61) | 4.36 (12.39) | 3.66 (12.17) | 7.68 (11.91) | 8.35 (10.82) | 8.35 (15.23) |
|  | Lower | 2.91 (9.9) | 3.55 (6.46) | 1.49 (8.46) | 7.96 (17.4) | 5.15 (10.38) | 7.94 (9.06) | 4.98 (8.47) |
| OxyHb changes at ch5 | | |  |  |  |  |  |  |
|  | Higher | 3.79 (13.9) | 4.51 (18.9) | -0.24 (20.74) | 1.17 (16.43) | 7.12 (13.31) | 9.36 (23.79) | 4.09 (20.40) |
|  | Lower | 4.26 (12.91) | 3.23 (10.31) | 2.65 (15.41) | 5.01 (20.69) | 7.39 (13.36) | 8.25 (11.96) | 7.06 (16.53) |
| OxyHb changes at ch6 | | |  |  |  |  |  |  |
|  | Higher | 3.70 (21.63) | 0.86 (16.35) | 3.95 (14.21) | -0.08 (18.36) | 8.84 (22.66) | 10.74 (23.12) | 11.32 (25.32) |
|  | Lower | 2.12 (17.1) | 5.94 (12.82) | 1.21 (12.00) | 3.80 (10.08) | 5.48 (17.61) | 10.22 (13.84) | 5.90 (12.26) |
| OxyHb changes at ch7 | | |  |  |  |  |  |  |
|  | Higher | 1.20 (9.42) | 4.02 (11.14) | 0.14 (15.72) | 4.77 (20.89) | 5.03 (9.03) | 8.02 (12.53) | 5.60 (15.97) |
|  | Lower | -0.23 (11.49) | 4.58 (11.98) | 1.48 (12.97) | 3.70 (9.65) | 3.08 (11.39) | 9.35 (15.90) | 7.40 (17.04) |
| OxyHb changes at ch8 | | |  |  |  |  |  |  |
|  | Higher | 11.63 (20.56) | 12.38 (24.67) | 13.61 (28.53) | 8.21 (29.26) | 16.95 (22.69) | 21.37 (29.98) | 20.66 (32.65) |
|  | Lower | 4.43 (16.11) | 4.87 (12.11) | 3.80 (16.67) | 5.02 (12.38) | 7.87 (18.11) | 8.89 (13.00) | 8.00 (16.84) |
| OxyHb changes at ch9 | | |  |  |  |  |  |  |
|  | Higher | 3.28 (8.1) | 1.72 (7.04) | 0.20 (12.89) | 0.21 (11.28) | 4.98 (8.14) | 4.17 (7.01) | 2.68 (12.99) |
|  | Lower | 0.23 (9.92) | 3.69 (11.93) | 1.69 (6.89) | 4.14 (10.61) | 2.84 (9.8) | 7.05 (12.12) | 5.44 (7.63) |
| OxyHb changes at ch10 | | |  |  |  |  |  |  |
|  | Higher | 7.55 (10.81) | 7.51 (10.04) | 9.43 (13.06) | 9.21 (14.54) | 10.08 (11.7) | 10.42 (10.92) | 13.46 (16.12) |
|  | Lower | 1.03 (12.93) | 2.72 (13.71) | 1.92 (10.38) | 4.15 (7.88) | 3.80 (12.32) | 7.01 (11.33) | 5.67 (9.15) |
| OxyHb changes at ch11 | | |  |  |  |  |  |  |
|  | Higher | 14.30 (20.81) | 12.38 (16.05) | 15.76 (19.8) | 12.65 (18.25) | 19.47 (24.69) | 18.47 (21.49) | 24.09 (26.62) |
|  | Lower | 1.13 (20.97) | 1.45 (14.52) | 2.02 (23.22) | 3.03 (17.34) | 0.83 (12.58) | 5.79 (13.16) | 2.76 (15.18) |
| OxyHb changes at ch12 | | |  |  |  |  |  |  |
|  | Higher | 5.48 (15.61) | 6.35 (15.97) | 3.86 (8.45) | 6.65 (14.57) | 9.14 (21.8) | 9.51 (19.24) | 6.99 (10.07) |
|  | Lower | 2.22 (11.23) | 5.42 (16.5) | 5.85 (13.88) | 6.04 (14.60) | 5.50 (11.49) | 8.72 (17.32) | 10.09 (19.19) |
| OxyHb changes at ch13 | | |  |  |  |  |  |  |
|  | Higher | 5.73 (10.96) | 4.02 (9.72) | 5.97 (9.02) | 2.74 (9.35) | 7.89 (11.55) | 7.12 (10.00) | 10.22 (12.01) |
|  | Lower | 0.76 (8.73) | 1.81 (10.2) | -0.48 (15.82) | 0.68 (16.37) | 4.16 (10.36) | 6.24 (11.60) | 3.85 (16.45) |
| OxyHb changes at ch14 | | |  |  |  |  |  |  |
|  | Higher | 5.76 (9.13) | 5.27 (14.01) | 6.33 (14.56) | 6.48 (16.72) | 8.89 (11.77) | 8.45 (15.49) | 10.43 (16.65) |
|  | Lower | 3.24 (11.15) | 3.17 (11.03) | 3.81 (9.92) | 3.90 (8.71) | 6.02 (12.24) | 6.64 (11.48) | 6.55 (10.88) |
| OxyHb changes at ch15 | | |  |  |  |  |  |  |
|  | Higher | 5.23 (12.01) | 5.03 (9.56) | 4.10 (9.05) | 1.88 (13.31) | 7.49 (12.12) | 7.64 (10.02) | 8.72 (12.21) |
|  | Lower | 2.97 (10.25) | 6.88 (11.14) | 2.81 (10.96) | 6.75 (9.91) | 5.91 (11.31) | 10.83 (13.73) | 6.74 (11.80) |
| OxyHb changes at ch16 | | |  |  |  |  |  |  |
|  | Higher | 4.94 (9.9) | 3.88 (14.71) | 5.71 (10.16) | 3.43 (17.55) | 7.54 (11.48) | 6.98 (16.03) | 11.04 (20.36) |
|  | Lower | 3.04 (19.16) | 5.07 (13.06) | 2.26 (17.21) | 6.15 (15.33) | 6.68 (21.81) | 9.43 (15.94) | 5.71 (19.18) |
| *Note*: RTs: response times, CVs: coefficient of variation, oxyHb: oxygenated hemoglobin, ch: channel. | | | | | | | | |


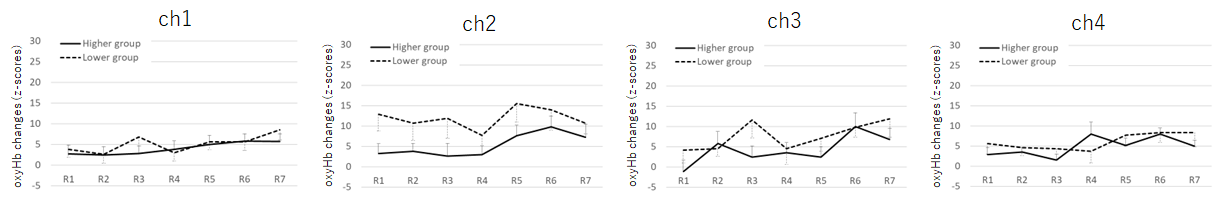


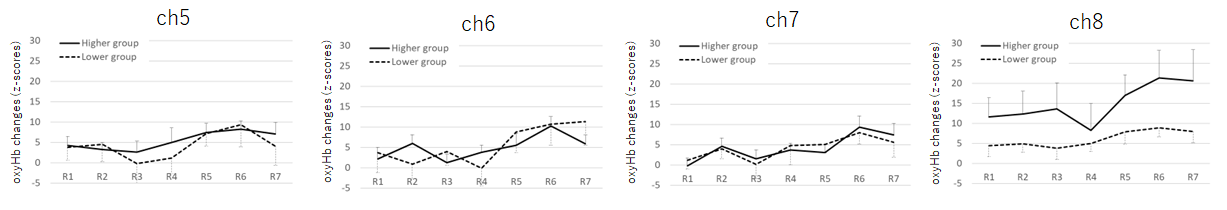


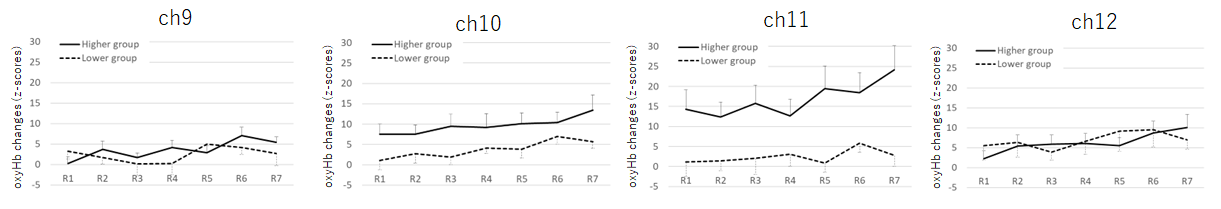


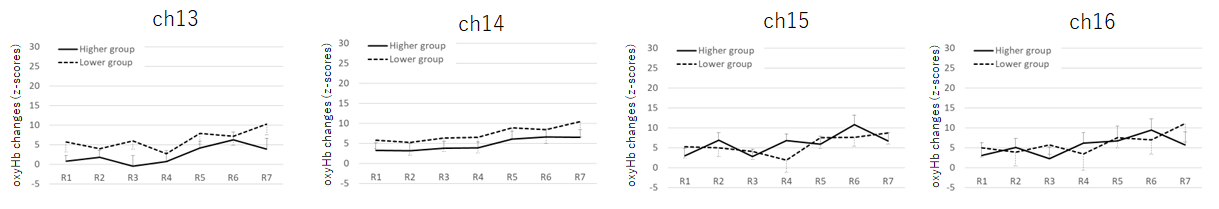


**Supplementary Figure 2.** Plots of oxyHb changes (z-scores) at all channels across each round during the SART for the higher and lower Cutaneous9-score groups. Error bars represent standard error. Ch, channel; SART, Sustained Attention to Response Tasks; oxyHb, oxygenated hemoglobin.
